# Discussions of Asperger Syndrome on Social Media: Content and Sentiment Analysis on Twitter

**DOI:** 10.2196/32752

**Published:** 2022-03-07

**Authors:** Elia Gabarron, Anders Dechsling, Ingjerd Skafle, Anders Nordahl-Hansen

**Affiliations:** 1 Department of Education, ICT and Learning Østfold University College Halden Norway; 2 Norwegian Centre for E-health Research University Hospital of North Norway Tromsø Norway; 3 Faculty of Health, Welfare and Organisation Østfold University College Kråkerøy Norway

**Keywords:** social media, autism spectrum disorder, health literacy, famous persons, Asperger, Elon Musk, twitter, tweets, mental health, autism, sentiment analysis

## Abstract

**Background:**

On May 8, 2021, Elon Musk, a well-recognized entrepreneur and business magnate, revealed on a popular television show that he has Asperger syndrome. Research has shown that people’s perceptions of a condition are modified when influential individuals in society publicly disclose their diagnoses. It was anticipated that Musk's disclosure would contribute to discussions on the internet about the syndrome, and also to a potential change in the perception of this condition.

**Objective:**

The objective of this study was to compare the types of information contained in popular tweets about Asperger syndrome as well as their engagement and sentiment before and after Musk’s disclosure.

**Methods:**

We extracted tweets that were published 1 week before and after Musk's disclosure that had received >30 likes and included the terms “Aspergers” or “Aspie.” The content of each post was classified by 2 independent coders as to whether the information provided was valid, contained misinformation, or was neutral. Furthermore, we analyzed the engagement on these posts and the expressed sentiment by using the AFINN sentiment analysis tool.

**Results:**

We extracted a total of 227 popular tweets (34 posted the week before Musk’s announcement and 193 posted the week after). We classified 210 (92.5%) of the tweets as neutral, 13 (5.7%) tweets as informative, and 4 (1.8%) as containing misinformation. Both informative and misinformative tweets were posted after Musk’s disclosure. Popular tweets posted before Musk’s disclosure were significantly more engaging (received more comments, retweets, and likes) than the tweets posted the week after. We did not find a significant difference in the sentiment expressed in the tweets posted before and after the announcement.

**Conclusions:**

The use of social media platforms by health authorities, autism associations, and other stakeholders has the potential to increase the awareness and acceptance of knowledge about autism and Asperger syndrome. When prominent figures disclose their diagnoses, the number of posts about their particular condition tends to increase and thus promote a potential opportunity for greater outreach to the general public about that condition.

## Introduction

### Background

Asperger syndrome (hereafter referred to as Asperger), which was removed from the Diagnostic and Statistical Manual of Mental Disorders, Fifth Edition (DSM-5) as a formal diagnosis, is currently merged with autism and pervasive developmental disorders that are otherwise not specified within “autism spectrum disorder” (hereafter referred to as autism). Although the International Classification of Diseases framework in its current 10th edition has not yet formally removed Asperger as a diagnosis; it will do so within the 11th version that is forthcoming [1]. However, those that have been diagnosed with Asperger may choose to use this as their formal diagnosis. The removal of Asperger from the DSM-5 has been controversial as many individuals took, and continue to take, pride in their Asperger identity [2]; however, many people with Asperger were also in favor of subsuming Asperger into the broader category of autism as a spectrum of conditions [3] on the grounds of, for instance, equality of service provision and legal protection. Although still a term used by many, Asperger is becoming more and more known as an integrated part of the broader autism spectrum and thus also a part of the blooming autistic neurodiversity movement [4]. Although less focus has been given to the term Asperger in recent years, public figures who are open about their diagnosis contribute to increased media attention. Examples of highly prominent people with Asperger who have been publicly open about their diagnosis include Elon Musk (who revealed his condition on Saturday Night Live on May 8, 2021) and Greta Thunberg (who refers to her Asperger diagnosis as her “superpower”). The media attention given to such disclosures typically leads to subsequent social media discussions that can inform but also misinform public perception [5]. Asperger and autism have become a part of pop culture [6,7], and research indicates there are advantages and disadvantages to this from a public health perspective but also, more importantly, an impact on individuals with the diagnosis. Asperger and autism are misunderstood and often “hidden.” They are hidden because there are no facial or bodily features that readily indicate a person has these conditions or not. Misunderstandings of the diagnoses relate directly to media representations (eg, films such as Rain Man [8] that are so influential that they can be perceived by the public as definitions for a whole diagnosis). For instance, savant syndrome is widely misconceived as very common in the population of individuals with autism and because of this and the intriguing features of the syndrome, it is a common part of media representation [9] of individuals with autism. However, the prevalence of savant syndrome in people with autism is rare [10]. Another widely held misconception of autism and Asperger is that people with autism, in general, are asocial [11]. Media attention is a double-edged sword in that public awareness and acceptance can increase, but it can also oversimplify highly complex heterogeneous conditions such as autism and Asperger. Oversimplifications and misconceptions can lead to stereotype thinking [12] and can maintain stigmas [13], but this could be reduced with the increase in societal awareness and understanding of the conditions [14]. This knowledge could be acquired or promoted through social media, where millions of people are exposed daily to all varieties of information, whether they explicitly seek it or find it unintentionally. Research on different conditions shows that health promotion through social media is mostly linked to positive effects [15-20].

Previous research has shown that the combination of key figures in society and their disclosures of personal diagnoses can affect the public’s perception of their conditions [21,22]. As with Musk’s disclosure and in the age of social media, the expectation was that the number of web-based discussions about Asperger and autism would increase, which could contribute further to spreading knowledge and awareness and thus potentially alter people’s perceptions of the condition.

### Objectives

The objective of this study was to compare the types of content used in (informative, misinformative, or neutral), the engagement with, and the sentiment of popular tweets about Asperger before and after Musk’s disclosure.

## Methods

### Study Design and Sample

We designed a cross-sectional study to analyze the type of content, engagement, and perception of popular tweets related to Asperger. We defined popular tweets as those with more than 30 likes as this is slightly higher than the average of 25 likes reported in previous research [23]. We focused on popular tweets because of their potential to reach more users.

### Eligibility Criteria for Tweets

We used the Twitter advanced search engine to find popular tweets (>30 likes) that were posted in English and included the terms “Aspergers” or “Aspie.” The term “Aspie” was included because it is a common slang term used by the autism community to refer to Asperger [5]. We extracted tweets that were published between May 1, 2021, and May 14, 2021, (1 week before and after Musk disclosed his Asperger diagnosis on May 8, 2021). As commonly used on other social media platforms, the “like” function on Twitter was chosen because of the quickness and ease (one click) with which it allows users to show their agreement with a posted tweet compared to the retweet function, which requires at least 2 clicks and potentially, the addition of accompanying text from the media user [24].

### Data Extraction and Classification

From the selected popular tweets, we extracted the post message and the number of comments, retweets, and likes. We extracted only the original tweets and no personal or identifiable data were collected.

We created a coding guideline, which was used to classify the tweets (the coding guideline is available in the data repository) [25]. Each post was classified by 2 independent coders who are experts in autism (IS and ANH) as to whether the post contained correct information (eg, “people with Asperger have difficulties in social interaction”), misinformation (eg, “people with Asperger have an IQ below average”), or neutral information (eg, “my son has Asperger”). Classification disagreements were discussed with the rest of the coauthors until a consensus was reached. We analyzed the number of posts and engagement from other media users with those posts. The engagement was assessed using the number of comments, retweets, and likes.

### Sentiment Analysis

We analyzed the perceptions or sentiments of each post using the AFINN sentiment analysis tool [26]. AFINN is a lexicon that assigns scores to each word ranging from –5 (very negative) to 5 (very positive) [25]. The text-mining tool considers scores higher than 0 as positive words, and scores lower than 0 as negative words [26]. The AFINN tool has been used to analyze the sentiment included in tweets about Asperger [5], and to compare the sentiment towards different COVID-19 vaccines expressed in social media posts [27]. The AFINN tool has also been used to analyze free short text, such as answers given in surveys [28,29], web-based reviews [30], self-reported notes [31], or descriptions of public health campaigns [32].

### Data Analysis

All statistical analyses were performed using SPSS (version 25.0; IBM Corp). Both the data set and data analysis, including scripts, were made available in the data repository [25]. The treatment of data for this study was approved by the data protection officer at the University Hospital of North Norway (Nr.02489).

## Results

We extracted a total of 227 popular tweets. A total of 34 (14.9%) tweets were posted the week before the Musk announcement and 193 (85%) were posted the following week.

### Engagement and Sentiment of Tweets Before and After Musk's Announcement

When we compared the engagement and sentiment of tweets before and after Musk's announcement, we found that tweets posted before the disclosure received significantly more engagement; they received more comments (254.15, 95% CI 87.1 to 331.5 compared to 44.88, 95% CI –74.6 to 493.2; *P*<.001), more retweets (494.47, 95% CI 28.3 to 635.6 compared to 190.80, 95% CI –321.6 to 928.9; *P*=.001), and more likes (7058.00, 95% CI 1734.9 to 10,443.6 compared to 969.44, 95% CI –4483.2 to 16,661.8; *P*<.001) than after the announcement (See Table 1). We did not find any statistically significant differences regarding the sentiment of tweets posted before and after Musk’s disclosure. Finally, the Kruskal-Wallis test did not show significant differences regarding engagement or sentiment according to the type of content provided by the tweet.

**Table 1:** Engagement with and sentiment of popular tweets about Asperger according to the time points when they were posted and the type of information provided.

Category of engagement	Time point when the tweet was posted		*t* test^a^	*P* value	Type of information provided			*P* value^b^
	Before Musk’s disclosure (n=34), mean (95% CI)	After Musk’s disclosure (n=193), mean (95% CI)			Provides information (n=13)^c^, mean (95% CI)	Neutral tweets (n=210)^d^, mean (95% CI)	Contains misinformation (n=4), mean (95% CI)	
Comments	254.15 (87.08 to 331.46)	44.88 (–74.63 to 493.16)	3.375	<.001	68.77 (–43.36 to 180.93)	77.96 (30.12 to 125.79)	9.50 (–0.77 to 19.77)	.81
Retweets	494.47 (–28.30 to 635.65)	190.80 (–321.57 to 928.91)	1.803	.001	146.66 (–47.94 to 340.86)	246.00 (117.76 to 374.23)	18.25 (–7.29 to 43.79)	.59
Likes	7058.00 (1734.96 to 10,443.57)	969.44 (–4483.23 to 16,661.76)	2.756	.001	824.00 (–216.54 to 1864.54)	1980.41 (277.11 to 3683.72)	124.75 (–26.96 to 276.46)	.26
Sentiment	0.12 (–1.42 to 1.08)	0.29 (–1.29 to 0.95)	0.272	.22	–1.46 (–3.20 to 0.28)	0.38 (–0.09 to 0.84)	0.00 (–2.60 to 2.60)	.21

^a^*df*=225.

^b^*P* value obtained from the Kruskal-Wallis test.

^c^Example of a tweet that provides information: “Not all autistic people (including people with Asperger’s diagnoses) are white, male techie types. Some of us are poets. Some of us are even women.”

^d^Example of a neutral tweet: “Elon Musk reveals he has Asperger’s syndrome during SNL monologue.”

### Informative, Neutral, and Misinformative Tweets Following Musk’s Announcement

The interrater agreement for the classification of the tweets and respective tweet data provided was κ=0.469 (moderate agreement). We classified 210 (92.5%) of the 227 tweets as being neutral, 13 (5.7%) tweets as informative, and 4 (1.8%) as containing misinformation. Both informative and misinformative tweets were posted after Musk’s disclosure. Tweets identified as misinformative included the following examples: tweets suggesting that Musk’s Asperger was deeply problematic, a tweet suggesting that autism and Asperger are the results of asymmetrical brain stem injuries, and a joke related to Musk’s development of the SpaceX Starship and the need of people with autism to travel into space.

## Discussion

### Summary of Findings

We found that after Musk's disclosure, the number of popular tweets about Asperger increased by almost 6-fold. This increase in tweeting has the potential to promote awareness of the Asperger condition to more social media users. However, these “after” tweets seemed to receive less engagement than the ones posted before the disclosure.

### Impact of a Celebrity Disclosure

As this was an observational study, the association between Musk´s disclosure and the increase in the number of tweets about Asperger receiving less engagement warrants further research to assess other possible factors affecting these results. It is nevertheless noteworthy that after Musk’s disclosure, discussions about Asperger on Twitter increased. On May 9, 2021, Asperger became the 19th top trending topic on Twitter [33]. The interest in Asperger syndrome was also reflected in Google searches, when on May 11, 2021, Asperger ranked as the top 28th trending search [34]. This is remarkable since neither of the terms “Aspergers” or “Autism” ranked in the top 50 trending search topics during World Autism Awareness Day (April 2, 2021).

With the increase of popular posts about Asperger, both informative and misinformative tweets also appeared. Although nonsignificant, the few popular tweets that did contain misinformation tended to receive less engagement than posts that provided neutral or informative content. The lower engagement with tweets containing misinformation could suggest a kind of collective intelligence among Twitter users [35] that could contribute to increasing social knowledge by reducing the spread of misinformation as seen with the autism tweets.

Considering the impact that celebrity culture has on directing the public’s attention to matters of health, it is worthwhile to investigate the types of information displayed on social media [36]. Within the field of autism research, very few studies assessing the informative and misinformative content posted on social media platforms have been conducted. This is slightly surprising since one of the more infamous studies, that resulted in a vast amount of misinformation on early childhood vaccination, was published (and later retracted) within this field. Misinformation about vaccines causing autism remains a problem today and is even propagated by some celebrities. Investigating the influence that celebrities, such as Musk, have on the public is underscored in our findings with the increase in attention that Asperger and autism received after Musk’s disclosure.

### Promoting the Awareness of Asperger and Autism Through Social Media

Promoting the awareness and acceptance of Asperger and autism by posting informative and factual content on social media platforms such as Twitter could assist in increasing social media users’ knowledge about and understanding of the condition. Research on the use of social media for health promotion has shown its positive effects related to different conditions [15-20]. However, in our sample, we found that the tweets on Asperger that stimulated higher engagement tended to be neutral. These neutral posts did not misinform, but they did not provide any type of information that could increase one’s knowledge or awareness about autism, either. Social media is ubiquitous and has become for most people a standard part of their everyday routine and habits. The ubiquitous use of social media platforms could provide an opportunity to expand the reach of trustworthy information about Asperger and autism in order to increase users’ knowledge about and awareness of the conditions and reduce the associated stigmas [5,14,21].

### Limitations

Our study had several limitations. We focused on a short period of time. We collected popular tweets about Asperger that were posted only in English. Furthermore, we only analyzed tweets posted the week after Musk’s announcement, which may not be enough to form a conclusion on the effect of the disclosure. Also, Twitter users may not be representative of a random sample of the population, as the platform’s users tend to range in age from 25 to 34 years [37]. Our findings may not apply to other social media platforms, to posts on autism that did not include our search terms, to posts in other languages, or to posts with less than 30 likes. We did not extract any identifiable information regarding the users that posted the popular tweets. Therefore, we cannot know if these tweets were posted by individuals or institutions. If posted by an institution, this could introduce a bias in terms of potential engagement impact (as institutions or organizations usually have more followers than individual accounts). Finally, due to the presence and sophistication of bot accounts, we cannot know if our sample size included tweets coming from any bot accounts [38].

### Conclusions

The use of social media platforms by health authorities, autism associations, and other stakeholders has the potential to increase awareness of and knowledge about both Asperger and autism. When prominent figures disclose their conditions, such as autism, posts about their condition tend to increase, which provides an opportunity for trustworthy information about the condition to reach more social media users. Future research on autism and celebrity engagement with media should deploy in-depth data collection and longitudinal designs to detect possible changes in sentiment, as well as different methodological approaches (eg, qualitative, quantitative, and mixed designs) to elucidate underlying mechanisms at play related to the spread of both information and misinformation.

## Abbreviations

DSM-5: Diagnostic and Statistical Manual of Mental Disorders, Fifth Edition

## References

[1] World Health Organization (2018). International statistical classification of diseases 11th revision: the global standard for diagnostic health information. World Health Organization.

[2] Huynh S, McCrimmon A, Strong T (2020). The change in classification of Asperger syndrome: an exploration of its effects on self-identity. Qual Rep.

[3] Kapp SK, Ne'eman A, Kapp SK (2020). Lobbying autism's diagnostic revision in the DSM-5. Autistic Community and the Neurodiversity Movement.

[4] Kapp SK (2020). Autistic Community and the Neurodiversity Movement: Stories From the Frontline.

[5] Skafle I, Gabarron E, Dechsling A, Nordahl-Hansen A (2021). Online attitudes and information-seeking behavior on autism, Asperger syndrome, and Greta Thunberg. Int J Environ Res Public Health.

[6] Dean M, Nordahl-Hansen A (2021). A review of research studying film and television representations of ASD. Rev J Autism Dev Disord.

[7] Hacking I (2009). How we have been learning to talk about autism: a role for stories. Metaphilosophy.

[8] Nordahl-Hansen A, Oien R, Volkmar FR (2017). Movie and TV depictions of autism spectrum disorder. Encyclopedia of Autism Spectrum Disorders.

[9] Nordahl-Hansen A, Tøndevold M, Fletcher-Watson S (2018). Mental health on screen: a DSM-5 dissection of portrayals of autism spectrum disorders in film and TV. Psychiatry Res.

[10] Skafle I, Nordahl-Hansen A, Øien RA (2019). Short report: social perception of high school students with ASD in Norway. J Autism Dev Disord.

[11] Howlin P, Goode S, Hutton J, Rutter M (2009). Savant skills in autism: psychometric approaches and parental reports. Philos Trans R Soc Lond B Biol Sci.

[12] Wood C, Freeth M (2016). Students’ stereotypes of autism. JEI.

[13] Yu L, Stronach S, Harrison AJ (2020). Public knowledge and stigma of autism spectrum disorder: comparing China with the United States. Autism.

[14] Morgan AJ, Ross A, Reavley NJ (2018). Systematic review and meta-analysis of Mental Health First Aid training: effects on knowledge, stigma, and helping behaviour. PLoS ONE.

[15] An R, Ji M, Zhang S (2017). Effectiveness of social media-based interventions on weight-related behaviors and body weight status: review and meta-analysis. Am J Health Behav.

[16] Dimanlig-Cruz S, Han A, Lancione S, Dewidar O, Podinic I, Haqani B, Haug J, Leonard L, Medline E, Patey A, Presseau J, Thompson E, Kent MP, Brouwers M (2021). Physical distancing messages targeting youth on the social media accounts of Canadian public health entities and the use of behavioral change techniques. BMC Public Health.

[17] Gabarron E, Wynn R (2016). Use of social media for sexual health promotion: a scoping review. Glob Health Action.

[18] Gabarron E, Årsand E, Wynn R (2018). Social media use in interventions for diabetes: rapid evidence-based review. J Med Internet Res.

[19] Hudnut-Beumler J, Po'e E, Barkin S (2016). The use of social media for health promotion in Hispanic populations: a scoping systematic review. JMIR Public Health Surveill.

[20] Simeon R, Dewidar O, Trawin J, Duench S, Manson H, Pardo Pardo J, Petkovic J, Hatcher Roberts J, Tugwell P, Yoganathan M, Presseau J, Welch V (2020). Behavior change techniques included in reports of social media interventions for promoting health behaviors in adults: content analysis within a systematic review. J Med Internet Res.

[21] Calhoun AJ, Gold JA (2020). “I feel like I know them”: the positive effect of celebrity self-disclosure of mental illness. Acad Psychiatry.

[22] Kresovich A, Noar SM (2020). The power of celebrity health events: meta-analysis of the relationship between audience involvement and behavioral intentions. J Health Commun.

[23] Indratmo Z, Buro K (2020). Comparisons between text-only and multimedia tweets on user engagement.

[24] Kaur W, Balakrishnan V, Rana O, Sinniah A (2019). Liking, sharing, commenting and reacting on Facebook: user behaviors’ impact on sentiment intensity. Telemat Inform.

[25] Gabarron E, Dechsling A, Skafle I, Nordahl-Hansen A (2021). Repository: Aspergers gaining momentum on social media. Zenodo.

[26] Nielsen FÅ (2011). A New ANEW: evaluation of a word list for sentiment analysis in microblogs.

[27] Marcec R, Likic R (2021). Using Twitter for sentiment analysis towards AstraZeneca/Oxford, Pfizer/BioNTech and Moderna COVID-19 vaccines. Postgrad Med J.

[28] Borakati A (2021). Evaluation of an international medical E-learning course with natural language processing and machine learning. BMC Med Educ.

[29] Denecke K, Gabarron E (2021). How artificial intelligence for healthcare look like in the future?. Stud Health Technol Inform.

[30] Compagner C, Lester C, Dorsch M (2021). Sentiment analysis of online reviews for selective serotonin reuptake inhibitors and serotonin-norepinephrine reuptake inhibitors. Pharmacy (Basel).

[31] Yoon S, Parsons F, Sundquist K, Julian J, Schwartz JE, Burg MM, Davidson KW, Diaz KM (2017). Comparison of different algorithms for sentiment analysis: psychological stress notes. Stud Health Technol Inform.

[32] Durand WM, Peters JL, Eltorai AEM, Kalagara S, Osband AJ, Daniels AH (2018). Medical crowdfunding for organ transplantation. Clin Transplant.

[33] (2021). Twitter trends on 9th May, 2021. Trend Calendar.

[34] Google trends on 11th May, 2021. Trend Calendar.

[35] Becker J, Brackbill D, Centola D (2017). Network dynamics of social influence in the wisdom of crowds. Proc Natl Acad Sci USA.

[36] Hoffman SJ, Tan C (2013). Following celebrities' medical advice: meta-narrative analysis. BMJ.

[37] Statista Distribution of Twitter users worldwide as of April 2021, by age group. 2021.

[38] Antonakaki D, Fragopoulou P, Ioannidis S (2021). A survey of Twitter research: data model, graph structure, sentiment analysis and attacks. Expert Syst Appl.

